# Quality of life at the dead sea region: the lower the better? an observational study

**DOI:** 10.1186/1477-7525-9-38

**Published:** 2011-05-27

**Authors:** Avital Avriel, Lior Fuchs, Ygal Plakht, Assi Cicurel, Armando Apfelbaum, Robert Satran, Michael Friger, Dimitry Dartava, Shaul Sukenik

**Affiliations:** 1 Pulmonary Unit, Soroka University Medical Center, Ben Gurion Avenue, Beer-Sheva, 84101, Israel; 2 Department of Internal Medicine "D", Soroka University Medical Center, Ben Gurion Avenue, Beer-Sheva, 84101, Israel; 3 Clinical Research Center, Soroka University Medical Center, Ben Gurion Avenue, Beer-Sheva, 84101, Israel; 4 Division of Community Health, Soroka University Medical Center, Ben Gurion Avenue, Beer-Sheva, 84101, Israel; 5 Department of Epidemiology, Faculty of Health Sciences, Ben-Gurion University of the Negev, Ben Gurion Avenue, Beer-Sheva, 84105, Israel

## Abstract

**Background:**

The Dead Sea region, the lowest in the world at 410 meters below sea level, is considered a potent climatotherapy center for the treatment of different chronic diseases.

**Objective:**

To assess the prevalence of chronic diseases and the quality of life of residents of the Dead Sea region compared with residents of the Ramat Negev region, which has a similar climate, but is situated 600 meters above sea level.

**Methods:**

An observational study based on a self-administered questionnaire. Data were collected from kibbutz (communal settlement) members in both regions. Residents of the Dead Sea were the study group and of Ramat Negev were the control group. We compared demographic characteristics, the prevalence of different chronic diseases and health-related quality of life (HRQOL) using the SF-36 questionnaire.

**Results:**

There was a higher prevalence of skin nevi and non-inflammatory rheumatic diseases (NIRD) among Dead Sea residents, but they had significantly higher HRQOL mean scores in general health (68.7 ± 21 vs. 64.4 ± 22, p = 0.023) and vitality (64.7 ± 17.9 vs. 59.6 ± 17.3, p = 0.001), as well as significantly higher summary scores: physical component score (80.7 ± 18.2 vs. 78 ± 18.6, p = 0.042), and mental component score (79 ± 16.4 vs. 77.2 ± 15, p = 0.02). These results did not change after adjusting for social-demographic characteristics, health-related habits, and chronic diseases.

**Conclusions:**

No significant difference between the groups was found in the prevalence of most chronic diseases, except for higher rates of skin nevi and NIRD among Dead Sea residents. HRQOL was significantly higher among Dead Sea residents, both healthy or with chronic disease.

## Introduction

The Dead Sea (DS) region has a unique climate. Its special therapeutic climatic advantages are recognized throughout the world. For many years this geographical area has served as a climatotherapy center for the treatment of various skin and rheumatic diseases [1-4], as well as pulmonary, cardiovascular, and gastrointestinal diseases [5-8].

The DS is situated in the Syrian-African Rift Valley. At 410 meters below sea level it is the lowest place in the world. Its geographic and meteorological characteristics generate a rare combination of climatic characteristics including:

1. The highest barometric pressure on earth (800 mm hg) with a partial oxygen pressure (PIO2) of 8% more than at sea level. This has therapeutic advantages in several respiratory and cardiovascular diseases [9,10].

2. A unique UV radiation, which is typical only of the DS region. UVB rays with a wavelength between 280-320 nanometers are differentiated from UVA rays with a wavelength between 320-400 nanometers. UVB waves cause the bulk of the skin damage (sun burns). The amount of radiation from both types of rays is reduced at the DS since they have to pass through an additional 420 meters to reach the ground. Furthermore, the high temperatures in the DS region cause significant evaporation of Dead Sea salts so the region has a sort of "vapor haze" that blocks radiation. The extent of blockage depends on the wavelength of the UV rays so that those with a shorter wavelength, UVB, are blocked more that UVA. Thus, the UVA/UVB ratio is higher at the DS than anywhere else in Israel and in the world.

3. A relatively low humidity (below 40%).

4. A paucity of rain (a few mm annually).

5. About 330 days of sunshine each year.

These unique environmental characteristics give the DS an advantage in the treatment of skin diseases such as psoriasis [11-13], atopic dermatitis [14], rheumatic diseases such as rheumatoid arthritis, psoriatic arthritis, ankylosing spondylitis, fibromyalgia and osteoarthritis [15-17], pulmonary diseases such as asthma [5] and COPD [10], and cardiovascular disease [6,18].

Previous studies assessed the climatic effects of the DS on patients with chronic diseases who came to the region for a short treatment period. The present study, in contrast to previously published studies, was a comparative study of the prevalence of chronic diseases and quality of life among DS residents and a control population of individuals who do not reside in the DS region, but in a region with a similar hot and dry desert climate, except for the differences in barometric pressure and UV radiation.

## Materials and methods

### Study population

The study population was comprised of kibbutz residents in the southern desert area of Israel. The kibbutz, a communal settlement based on socialist ideology, is among the most economically homogeneous societies in the western world. The study group consisted of residents of five kibbutzim in the DS region. These kibbutzim were selected as the main settlements with permanent inhabitants of the region. The control group consisted of residents of two kibbutzim in the Ramat Negev (RN) region, which is also in the southern desert, just 100 km from DS area, and also has a hot and dry desert climate. These *two*****Kibbutzim were selected in order to have as homogeneous a control group as possible. Both regions are sparsely populated. In contrast to the DS region, the RN region is located in the mid-Negev heights. It is the highest region in the Negev desert located about 600 meters above sea level with barometric pressure of 710 mmHg. The mean annual rainfall in this region is 100 mm compared to few mm at the DS region.

The participants in both groups had similar socioeconomic, cultural, ethnic and occupational backgrounds [19].

Inclusion criteria were residents of the two regions, above the age of 18, who agreed to complete the questionnaire. Individuals who resided in the two regions for less than a year were excluded from the study.

### Study design

Study participants completed a structured self-administered questionnaire that was distributed at primary care clinics and via the kibbutz internal mail system. Participation was on a voluntary basis. The questionnaire had two parts. The first part assessed baseline characteristics including socio-demographic variables such as age, family status, place of work (indoors or outdoors), life habits (tobacco and/or alcohol), and chronic diseases. The participants had to indicate the presence or absence of chronic diseases from a list of chronic diseases.

The second part was a validated SF-36 questionnaire of the Medical Outcomes Study (MOS), to assess health-related quality of life (HRQOL). The SF-36 questionnaire contains 36 items measuring health across eight areas or domains: Physical Functioning (PF) 10 items; Social Functioning (SF) 2 items; Role Limitations due to physical problems (RP) 4 items; Role Limitations due to emotional problems (RE) 3 items; Mental Health (MH) 5 items; Vitality (VT) 4 items; Bodily Pain (BP) 2 items and General Health perceptions (GH) 5 items. There is also an additional item on perceived changes in health status over the past 12 months. Four scales (PF, RP, BP and GH) make up the Physical Component Summary (PCS) measure and the other four scales (VT, SF, RE and MH) make up the Mental Component Summary (MCS). Scores are coded for each dimension, summed and transformed to generate a score from 0 (worst possible health state) to 100 (best possible health state) [20-22]. The SF-36 has proven useful in surveys of general and sick populations, comparing the relative burden of diseases, and in differentiating the health benefits produced by a wide range of different treatments [23]. The SF-36 Health survey has been translated to and validated in Hebrew [24].

The study was approved by the Helsinki committee of Soroka University Medical Center, Beer-Sheva.

### Statistical analysis

The baseline characteristics were compared between the two study groups using the Chi-square and t-tests. Multivariate analyses, using a logistic regression model, were conducted to compare the prevalence of the investigated chronic diseases, adjusted for demographics and health-related habits. Comparisons of the HRQOL components was performed with Mann-Whitney U test, and for multivariate analysis linear regression models were computed [25]. The dependent variables in these models were the HRQOL scales. The independent variables for the models were demographics, health-related habits and chronic conditions. A p-value lower than 0.05 was considered significant for all statistical analyses.

## Results

Three hundred three of 730 residents from the DS (45%) region completed the study questionnaire compared to 251 of 710 (35%) from the RN region.

Table 1 summarizes the socio-demographic and chronic disease data for the two study groups. DS inhabitants were younger, with a lower percentage of married participants and a higher percentage of participants who worked outdoors.

**Table 1:** Comparison of socio-demographic variables, health-related habits, and chronic diseases between the study groups.

Variable	DS (n = 303)	RN (n = 251)	p-value
**Age **(mean ± SD)	44.7 ± 14.7	53.1 ± 17.5	< 0.001
**Gender **(% female)	55.6	61.8	0.138
**Family status **(%)			
Single	19.5	11.6	0.002
Married	65.2	73.3	
Divorced	12.5	8	
Widowed	2.7	6.8	
**Health-related habits **(%)			
Works outdoors	35.9	20.3	< 0.001
Smokes	23.3	23.1	0.949
Consumes alcohol	2.7	1.2	0.198
**Chronic co-morbidity **(%)			
Heart disease	5.2	6.8	0.409
Asthma	5.2	4.8	0.839
Other chronic lung disease	4.2	3.6	0.688
Malignancy	7.9	9.2	0.581
Stroke	0.6	2	0.129
Diabetes mellitus	6.4	6.4	0.996
Hypertension	17.6	17.5	0.989
Psychiatric disease	4.2	3.6	0.688
Inflammatory bowel disease	0.6	0.4	0.729
**Skin disease**			
Inflammatory	5.5	4.8	0.005
Skin nevi	15.5	6.8	
**Rheumatic disorders**			
Inflammatory	2.1	4	0.15
Non-inflammatory	30.6	24.7	
Vascular disease	8.2	12.4	0.097

DS = Dead Sea group

RN = Ramat Negev group

The univariate analyses showed no significant difference in the prevalence of most chronic diseases between the two groups, except for a significantly higher percentage of skin nevus (p = 0.008) and non-inflammatory rheumatic diseases (NIRD) (p = 0.028) in the DS group (Table 2).

**Table 2:** Comparative multivariate analysis of risk for chronic diseases between the study groups*.

Variable	OR (95%CI) Unadjusted	p-value	OR (95%CI) Adjusted**	p-value
Heart disease	0.75 (0.37;1.50)	0.409	1.65 (0.60;4.49)	0.328
Asthma	1.08 (0.51;2.31)	0.839	0.80 (0.29;2.20)	0.661
Other chronic lung disease	1.19 (0.51;2.80)	0.688	1.31 (0.41;4.18)	0.649
Malignancy	0.85 (0.47;1.52)	0.581	1.24 (0.56;2.72)	0.596
Stroke	0.30 (0.05;1.56)	0.129	0	
Diabetes mellitus	1.0 (0.51;1.96)	0.996	1.16 (0.49;2.68)	0.73
Hypertension	1.0 (0.65;1.55)	0.989	2.87 (1.48;5.55)	0.002
Psychiatric disease	1.19 (0.51;2.80)	0.688	2.12 (0.76;5.92)	0.15
Inflammatory bowel disease	1.52 (0.14;16.91)	0.729	0.72 (0.035;14.49)	0.828
**Skin disease**				
Inflammatory	1.15 (0.54;2.43)	0.716	1.09 (0.46;2.58)	0.842
Skin nevi	2.52 (1.42;4.48)	0.001	2.49 (1.27;4.90)	0.008
**Rheumatic disorders**				
Inflammatory	0.55 (0.20;1.39)	0.187	0.88 (0.29;2.71)	0.83
Non-inflammatory	1.34 (0.93;1.95)	0.117	1.69 (1.06;2.69)	0.028
Vascular disease	0.63 (0.37;1.09)	0.097	0.92 (0.46;1.86)	0.824

* For all comparisons, Ramat Negev group used as the reference group.

**Adjusted for socio-demographic variables and health-related habits.

Of the 69 DS participants who reported skin disease, 33% were treated with oral drugs and skin creams, compared to 69% of 29 corresponding participants in the control group (p = 0.001).

**Figure 1:**
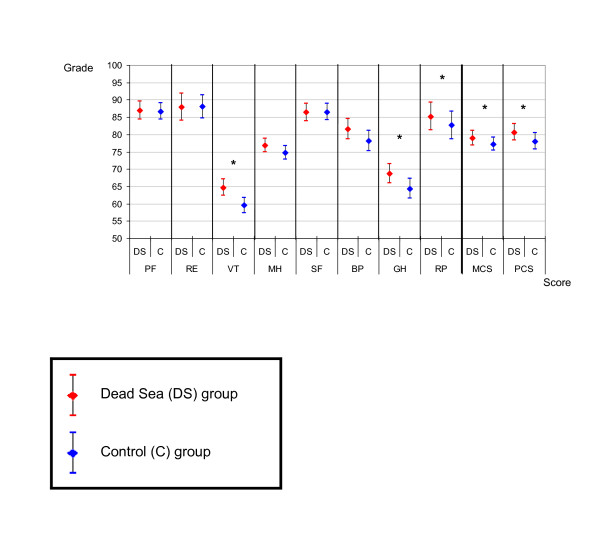
**Comparing SF-36 quality of life scores (Mean and 95% Confidence Interval) between DS inhabitant and control group**. PF - Physical Function (p = 0.387). RE - Role-Emotional (p = 0.560). VT - Vitality (p = 0.001). MH - Mental Health (p = 0.152). SF - Social Function (p = 0.868). BP - Bodily Pain (p = 0.071). GH - General Health (p = 0.023). RP - Role-Physical (p = 0.245). MCS - Mental Component Summary (p = 0.020). PCS - Physical Component Summary (p = 0.042). * Significant difference of scores between Dead See and Control groups (p < 0.05).

HRQOL scores were significantly higher among DS residents in the GH (68.7 ± 21 vs. 64.4 ± 22, p = 0.023) and VT (64.7 ± 17.9 vs. 59.6 ± 17.3, p = 0.001) categories, and in the summary measures: PCS (80.7 ± 18.2 vs. 78 ± 18.6, p = 0.042), and MCS (79 ± 16.4 vs. 77.2 ± 15, p = 0.02) (Figure 1).

After adjustment for demographics (including age differences), health-related habits and chronic diseases, the difference in HRQOL increased. The DS residents had higher HRQOL scores in more categories (including VT, BP, GH, and RP) as well as in the PCS and MCS summary measures (Table 3).

**Table 3:** Comparative multivariate analysis of SF-36 quality of life scores between the study groups*.

SF-36 scale	Regression Coefficient (B)	Standard Error of B	Standardized Regression Coefficient (Beta)	p-value
Physical function (PF)	1.35	1.77	0.04	0.448
Role emotion (RE)	1.8	2.82	0.03	0.524
Vitality (VT)	6.78	1.78	0.19	<0.001
Mental health (MH)	2.59	1.51	0.09	0.088
Social function (SF)	2.64	1.83	0.07	0.151
Bodily pain (BP)	5.75	2.2	0.13	0.009
General health (GH)	4.26	2.13	0.1	0.046
Role physical (RP)	7.4	2.89	0.13	0.011
Mental component summary (MCS)	3.94	1.54	0.13	0.011
Physical component summary (PCS)	4.3	1.66	0.12	0.01

* For all comparisons, Ramat Negev group used as the reference group. Adjusted for socio-demographic variables and health-related habits.

## Discussion

The main purpose of the study was to assess whether there is a difference in the prevalence and severity of chronic diseases, as well as HRQOL, between residents of an area below sea level with a unique elevated barometric pressure and a unique solar spectrum of UV light, and residents of an area above sea level.

The DS and RN regions are both sparsely populated, dry desert areas in the southern part of Israel. However, the DS is the lowest place in the world and has unique geographical and meteorological characteristics that create a rare combination of climate conditions that are considered conducive to health and HRQOL.

The results of previous studies have demonstrated the advantage of the DS region for climatotherapy. Most of these studies examined the health benefits of the DS region for patients with chronic diseases who came to the DS for treatment. None of these studies assessed permanent residents of the DS region to determine whether the affects of this unique climate are beneficial to residents of the region in terms of the prevalence of chronic diseases and HRQOL. Thus, for the first time, the study group consisted DS region residents who were compared with a control group of individuals who live in the same desert area of southern Israel, but at a much higher altitude, above sea level and without the unique climate characteristics of the DS.

Although there were similar ethnic and socioeconomic characteristics between the study groups, the DS population was younger, had fewer married participants, and was more likely to work outdoors (the last two variables may be related to the age differences). After adjusting for these variables (including age difference) we still found differences between the groups in the prevalence of NIRD and skin nevus. We cannot determine, on the basis of the study data, whether this increased prevalence reflects an influx of individuals with chronic diseases to the DS region in the belief that it has a favorable effect on their disease, or that the DS climate only has therapeutic, not preventive, properties. We did not find any national immigration data or scientific literature showing a trend for people with chronic disease to immigrate to known climatotherapy areas, but this is an issue that should be investigated further.

Another finding was that DS region residents with skin disease use less oral medication and/or skin cream. This might be because their skin disease is less severe in this region because of its beneficial climatotherapeutic effect. Studies published over the past 40 years [13,14,26,27] have shown that the DS region has a significant climatotherapeutic effect on skin disease (psoriasis, atopic dermatitis, vitiligo, acne, mycosis fungoides and psoriatic arthritis), but there have been no previous reports of skin nevi among the DS residents. Our finding is surprising in light of the region's unique UV radiation filtration. The carcinogenic effect of sun exposure and other environmental factors that can cause pre-malignant or malignant skin lesions in DS region residents has not been studied. There have been studies of a late carcinogenic sun exposure risk in patients with skin disease treated for non-malignant skin conditions by therapeutic exposure to the sun. The results of these studies are non-conclusive or controversial [28-30]. A recently published study [31] showed that sun exposure in the DS was not associated with an increased risk of skin cancer or melanoma, but contended that UV radiation exposure at the DS region may play a role in the development of skin damage. Another study [32] recommended reduction of the amount of daily therapeutic DS sun exposure to get the same therapeutic effect with decreased risk of damage. We cannot determine, on the basis of the present results, whether the higher prevalence of skin nevi is due to environmental factors in the DS region or can be attributed to a tendency of DS region residents to take fewer protective measures due to a common, but mistaken, belief that they are protected in this region.

Residents in the DS region, both healthy and with chronic disease had significantly higher HRQOL measures than residents in the RN region. The difference was even stronger after adjustment for socio-demographic variables and chronic diseases. It is not clear from the results of the present study whether this difference is due to the climatotherapeutic characteristics of the DS region, or to other non-biological environmental characteristics. Previous studies showed that the DS region has a beneficial therapeutic effect on patients with chronic diseases who came the DS as health tourists [33,34]. These studies demonstrated reduced pain, improved strain and physical task performances, improved energy and general health parameters, and improved emotional and social parameters after the stay in the DS region.

However, these studies were conducted on patients who came to the DS region for recreational as well as therapeutic purposes, which may cause a methodological bias in terms of improved HRQOL. In the present study we examined the DS region's effects on residents who have lived and worked there for a long time. The results, which demonstrate higher HRQOL measures for healthy and chronically ill residents, reinforce the results of previous studies that the DS region has potent climatotherapeutic effects.

Potential limitations of this study include its small sample size. Although more than 40 percent of the population responded to our questionnaire forms, the participating kibbutzim had a total population of only several hundreds residents with relatively few chronically ill patients. As participation in this study was on a voluntary basis we cannot be sure that the enrolled individuals are totally representative of the entire region population.

In this study we did not assess the clinical severity of the patients' chronic diseases. One could argue that the fact that many HRQOL parameters are better in the DS region is an indirect marker of less severe and disabling chronic disease in the region, but we cannot prove this assumption based on the results of the study.

Also, the data cannot determine whether the climate at below sea level caused the higher prevalence of skin nevi and NIRD.

We tried to assess changes in disease severity in chronically ill patients who live in the DS area, but stayed for a period of time in places above sea level. However, we were not able to draw any conclusions because of a very low response rate.

Future prospective studies should assess the clinical characteristics of different chronic diseases and compare their course, severity, and clinical outcome between residents of the DS region and other comparison populations.

We conclude that HRQOL is significantly higher among both healthy and chronically ill residents of the DS region compared with residents of the control group region, although more residents in the region have skin nevi and NIRD.

## Competing interests

The authors declare that they have no competing interests.

## Authors' contributions

AA, LF and SS - Study design, study coordinators, data collection and data processing, writing of article.

YP, MF - Statistics.

AC, AA, RS, DD - Family physicians, patients recruitment and questionnaires distribution and collection.

All authors read and approved the final manuscript.
